# Duration of Parathyroid Function Recovery in Patients With Protracted Hypoparathyroidism After Total Thyroidectomy for Papillary Thyroid Carcinoma

**DOI:** 10.3389/fendo.2021.665190

**Published:** 2021-04-19

**Authors:** Yuxuan Qiu, Zhichao Xing, Qiao Xiang, Qianru Yang, Anping Su, Yan Luo

**Affiliations:** ^1^ Department of Ultrasound, West China Hospital, Sichuan University, Chengdu, China; ^2^ Center of Thyroid and Parathyroid Surgery, West China Hospital, Sichuan University, Chengdu, China; ^3^ Department of Endocrinology and Metabolism, West China Hospital, Sichuan University, Chengdu, China

**Keywords:** parathyroid function recovery, protracted hypoparathyroidism, permanent hypoparathyroidism, total thyroidectomy, papillary thyroid carcinoma

## Abstract

**Purpose:**

The aim of the present study is to investigate the time to recovery of parathyroid function in patients with protracted hypoparathyroidism at 1 month after total thyroidectomy of papillary thyroid carcinoma.

**Materials and Methods:**

Adult patients who underwent total thyroidectomy for papillary thyroid cancer were included. Cases of long-term hypoparathyroidism were studied for recovery of parathyroid function during the follow-up. The duration of recovery and associated variables were recorded.

**Results:**

Out of the 964 patients, 128 (13.28%) developed protracted hypoparathyroidism and of these, 23 (2.39%) developed permanent hypoparathyroidism and 105 (10.89%) recovered: 86 (8.92%) before 6 months, 11 (1.14%) within 6 and 12 months and 8 (0.83%) after 1 year follow-up. Variables significantly associated with the time to parathyroid function recovery were number of autotransplanted parathyroid glands (HR, 1.399; 95% CI, 1.060 – 1.846; *P* = 0.018), serum calcium concentration >2.07 mmol/L (Hazard ratio [HR], 1.628; 95% confidence interval [CI], 1.009 – 2.628; *P* = 0.046) and PTH level > 1.2 pmol/L (HR, 1.702; 95% CI, 1.083 – 2.628; *P* = 0.021) at 1 month postoperatively.

**Conclusion:**

Permanent hypoparathyroidism should not be diagnosed easily by time, since up to one-fifth of the patients will experience recovery after a period of 6 months and a few patients even beyond one year. The number of autotransplanted parathyroid glands is positively associated with the time to parathyroid function recovery.

## Introduction

The most common risk after a total thyroidectomy for differentiated thyroid cancer is transient hypoparathyroidism caused by either inadvertently removed or devascularized parathyroid glands during the surgery (1). Most patients with hypoparathyroidism regain parathyroid function within 1 month after surgery; however, in some patients, the hypoparathyroidism may persist over time. Permanent hypoparathyroidism represented the last stage of protracted hypoparathyroidism, but its accurate incidence was highly variable (2).

There is still a lack of consensus regarding the follow-up period necessary to make a firm diagnosis of permanent hypoparathyroidism. The European Guidelines defined permanent hypoparathyroidism as low serum parathyroid hormone (PTH) levels and/or need for replacement therapy after six months after surgery (3), and the American Thyroid Association chose six months as the cut-off time as well (4). However, the American Association of Clinical Endocrinologists extended the follow-up period to one year to diagnose permanent hypoparathyroidism (5). During this period, the main goal of the treatment is to maintain serum calcium within the reference range to avoid symptoms of hypocalcemia and to promote restoration of parathyroid function. There is little empirical evidence to determine the follow-up time required to determine complete loss of parathyroid function after thyroidectomy, because most studies on the recovery of the parathyroid function are limited in sample size and/or report a short observation time (6, 7). Once a diagnosis of permanent hypoparathyroidism is made, it implies lifelong medical care and financial burden (8). More importantly, hypoparathyroidism related complications such as decrease in renal function and higher risk for cardiovascular diseases, not just hypoparathyroidism itself, would highly impair the quality of life (9, 10).

Hence, in consideration of its clinical relevance, we conducted this study determine the incidence and period of recovery of parathyroid function in patients with protracted hypoparathyroidism and to identify variables associated with delayed restoration of parathyroid function.

## Method

This study is presented in accordance with the STROBE (STrengthening the Reporting of Observational studies in Epidemiology) checklist.

### Study Design

An observational study was performed in a cohort of consecutive patients who underwent total thyroidectomy for papillary thyroid carcinoma at the Center of Thyroid & Parathyroid Surgery, between July 2013 and June 2017 where only a single experienced team performs thyroid surgery. Eligibility criteria were adult patients (≥18 years old) who received primary total thyroidectomy for papillary thyroid cancer. Exclusion criteria were associated parathyroidectomy, completion thyroidectomies and recurrent cancers. The main outcomes of the study were the prevalence of permanent hypoparathyroidism and the time to recovery of parathyroid function. The secondary objective was to investigate factors influencing early (≤6 months) or late (>6 months) recovery of parathyroid function. Informed consent for thyroidectomy and use of anonymized data for clinical research was obtained.

### Surgical Procedure

The indications for total thyroidectomy were according to the 2015 American Thyroid Association Guidelines (11). Bilateral central neck dissection was applied for 1) bilateral PTC; 2) isthmus PTC; 3) stage T3 and T4 PTC; 4) prelaryngeal and/or pretracheal lymph node metastases; 5) bilateral central lymph node or lateral lymph node metastases; and 6) TERT promoter mutation (confirmed by preoperative fine-needle aspiration biopsy) (12). Otherwise, unilateral central neck dissection would be adopted according to the intraoperative situation. Therapeutic lateral neck dissection was performed as clinically indicated.

Carbon nanoparticles were used for better identification of parathyroid glands during the operation. Generally, thyroid or lymph tissues can be staining black by carbon nanoparticles while parathyroid glands cannot because of their different lymphatic drainage (13). It is reported that use of carbon nanoparticles could help preserve parathyroid glands *in situ* and decrease transient hypoparathyroidism rate (14, 15). Every attempt was made to carefully preserve each parathyroid gland with its blood supply to ensure its function. When a parathyroid gland was devascularized in the surgical field or resected unintentionally, it would be autotransplantated to the contralateral sternocleidomastoid muscle after confirmation by intraoperative frozen biopsy.

### Perioperative Management

Perioperative management of all patients was standardized. Preoperative examinations included serum PTH levels, calcium concentrations, thyroid function, neck ultrasound, and laryngoscopy. Prophylactic calcium supplementation (calcium gluconate 4g, intravenous drip) was given once to each patient immediately after surgery. Serum PTH and calcium levels were then regularly tested on the next day (24 hours after surgery). If tested serum PTH and calcium levels were within the normal range (PTH, 1.6-6.9 pmol/L; calcium, 2.1-2.7 pmol/L), no additional supplementation would be arranged. IF tested serum PTH and calcium levels were below the normal range, extra oral administration of 1.5–3 g/day of calcium carbonate and 0.5–1.5 μg/day of calcitriol would be given. If a sudden symptom of hypocalcemia occurred, an emergency intravenous drip of calcium supplementation would be added.

### Follow-Up

All the patients were first followed at one month after surgery. Patients with serum PTH levels detected below the normal range were followed thereafter until their parathyroid function recovered or permanent hypoparathyroidism was diagnosed. Recovery of parathyroid function was defined as a normal value for PTH in asymptomatic patients not requiring replacement therapy. Permanent hypoparathyroidism was diagnosed if serum PTH was lower than normal range at the last follow-up point and replacement therapy was still required. In brief, patients with protracted hypoparathyroidism at one month were followed indefinitely until recovery of parathyroid function or permanent hypoparathyroidism was diagnosed. Weaning off replacement therapy after one month was initiated when serum calcium concentration and/or PTH became normal or showing a sustained increase.

### Statistical Analysis

A univariate analysis was performed comparing patients who recovered or did not recover from protracted hypoparathyroidism, and those who recovered before or after six months. Continuous and normally distributed variables were expressed as mean ± standard deviation (SD) and compared using student’s t-test for unpaired samples. Non-normally distributed variables were compared using the Mann–Whitney U test. Dichotomous variables were compared using the χ^2^ test, continuity correction of χ^2^ test or Fisher’s exact test as appropriate. The best cut-off values of continuous variables were intercepted from the receiver operating characteristic (ROC) curve with the largest aera under curve (AUC) to further differentiate the between-group differences. Binomial logistic regression analysis with predictors selected by a forward stepwise procedure was used to assess the risk factors for permanent hypoparathyroidism. Time to recovery of parathyroid function was analyzed using Kaplan–Meier survival curves and the Log Rank (Mantel-Cox) test. The statistical analysis was carried out using Stata/SE 15.1 (StataCorp LLC). Statistical significance was set at P < 0.05.

## Results

### Follow-Up Outcomes

**Figure 1:**
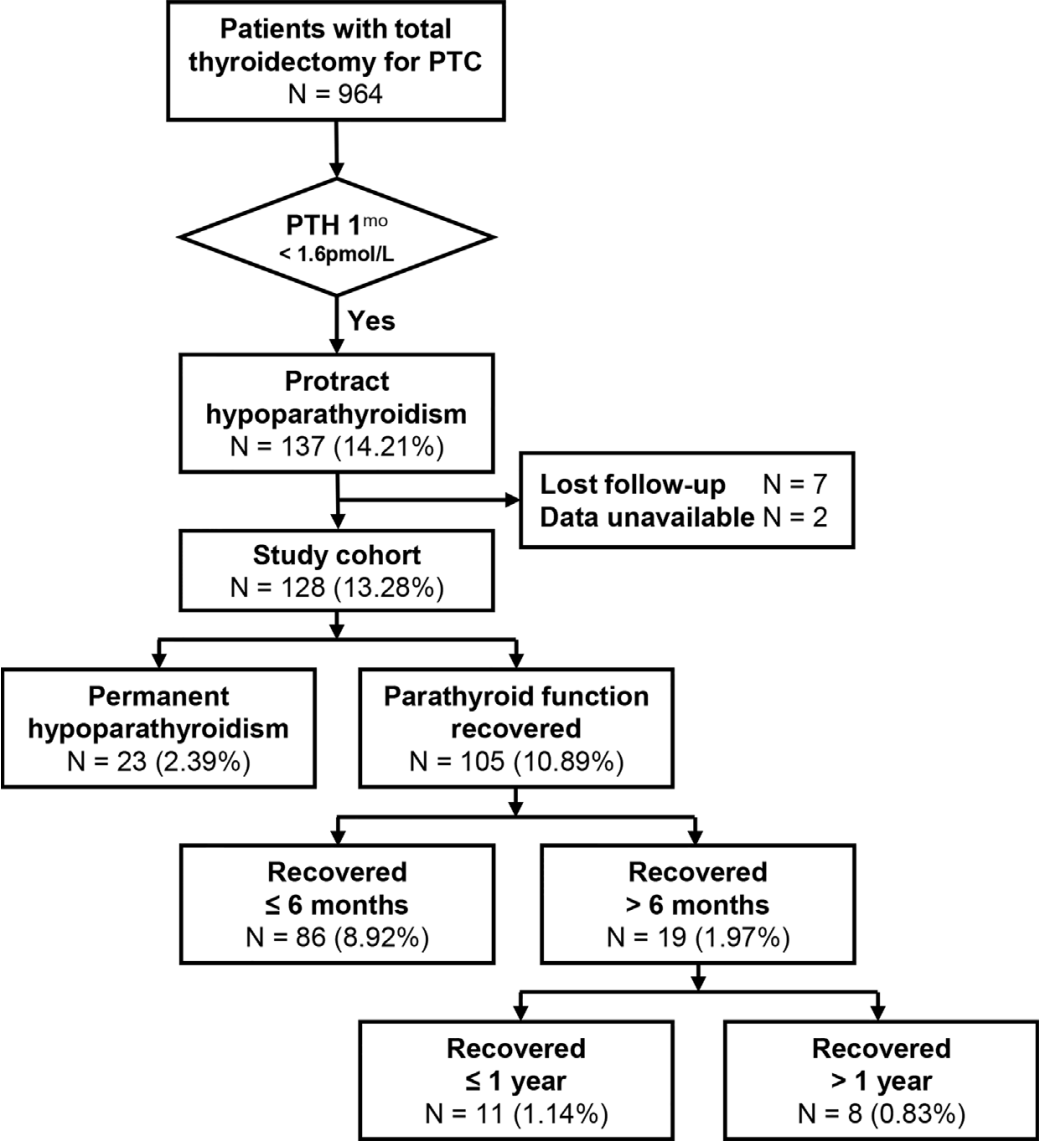
Patient flow diagram. Highlighted area encloses the study patients. Percentages are over the immediately preceding group. PTH, parathormone; 1^mo^, 1 month postoperatively.

The flow chart of patients included in the study was shown in **Figure 1**. The initial cohort enrolled 964 patients, and 128 (13.28%) patients with protracted hypoparathyroidism were finally included in this study. A total of 23 (2.39%) had permanent hypoparathyroidism while 105 (10.89%) recovered parathyroid function during the follow-up, in which 86 (8.92%) recovered within 6 months and 19 (1.97%) recovered beyond this time point.

### Recovery of Parathyroid Function and Permanent Hypoparathyroidism

The baseline characteristics and surgical or laboratory variables between patients who recovered or had permanent hypoparathyroidism were shown in **Tables 1** and **2**, respectively. Patients≤ 55 years old or without hypertension were more likely to recover parathyroid function. Carbon nanoparticles or parathyroid autotransplantation were more frequently used in patients recovering parathyroid function. No early biochemical parameter was predictive of the long-term parathyroid function. At one month postoperatively, patients who recovered parathyroid function were higher in serum calcium and lower in serum phosphorus levels. Both normal–high serum calcium levels (>2.07 mmol/L) and serum PTH concentrations (>1.2 pmol/L) were associated with higher proportions of recovery of parathyroid function. The multivariate analysis identified that use of carbon nanoparticles and serum calcium level >2.07 mmol/L and PTH > 1.2 pmol/L at 1 month could independently predict recovery of parathyroid function (**Table 3**).

**Table 1:** Demographics of patients with protracted hypoparathyroidism who recovered the parathyroid function or developed permanent hypoparathyroidism.

Variables	Recovered N = 105 (%)	Permanent N = 23 (%)	*P*
Gender			0.832
Male	25 (23.8)	5 (21.7)	
Female	80 (76.2)	18 (78.3)	
Age, years	41.2 ± 12.0	45.7 ± 12.8	0.108
≤55	94 (89.5)	17 (73.9)	0.046
>55	11 (10.5)	6 (26.1)	
BMI	22.65 ± 3.5	23.28 ± 3.38	0.426
Hypertension			0.042
No	97 (92.4)	18 (78.3)	
Yes	8 (7.6)	5 (21.7)	
Diabetes*			1.000
No	102 (97.1)	23 (100)	
Yes	3 (2.9)	0 (0)	
Hashimoto’s thyroiditis			0.276
No	80 (76.2)	15 (65.2)	
Yes	25 (23.8)	8 (34.8)	
Hypothyroidism**			0.475
No	102 (97.1)	21 (91.3)	
Yes	3 (2.9)	2 (8.7)	
^131^I ablation			0.694
No	41 (39.0)	10 (43.5)	
Yes	64 (61.0)	13 (56.5)	
T stage			0.780
T1a	27 (25.7)	8 (34.8)	
T1b	33 (31.4)	6 (26.1)	
T2	6 (5.7)	3 (13.0)	
T3a	1 (1.0)	0 (0)	
T3b	24 (22.9)	4 (17.4)	
T4a	12 (11.4)	2 (8.7)	
T4b	2 (1.9)	0 (0)	
N stage			0.825
N0	48 (45.7)	10 (43.5)	
N1a	27 (25.7)	5 (21.7)	
N1b	30 (28.6)	8 (34.8)	

Continuous variables given as mean ± standard deviation; *Fisher’s test.; ** χ^2^ test with continuity correction.

**Table 2:** Surgical and laboratory variables of patients with protracted hypoparathyroidism who recovered the parathyroid function or developed permanent hypoparathyroidism.

Variables	Recovered N = 105 (%)	Permanent N = 23 (%)	*P*
Carbon nanoparticles			0.020
No	25 (23.8)	11 (47.8)	
Yes	80 (76.2)	12 (52.2)	
Central neck dissection			0.590
No	22 (21.0)	6 (26.1)	
Yes	83 (79.0)	17 (73.9)	
Central neck dissection (Yes)			0.418
Unilateral	17 (20.5)	5 (29.4)	
Bilateral	66 (79.5)	12 (70.6)	
Lateral neck dissection			0.856
No	71 (67.6)	16 (69.6)	
Yes	34 (32.4)	7 (30.4)	
No. of autotransplanted parathyroid glands			0.028
0	22 (21.0)	11 (47.8)	
1	56 (53.3)	8 (34.8)	
2-3	27 (25.7)	4 (17.4)	
Serum calcium (Ca, mmol/L)			
Ca preop	2.32 ± 0.09	2.32 ± 0.13	0.884
Ca^24h^ postop	2.02 ± 0.20	1.99 ± 0.15	0.548
Ca^1mo^ postop	2.22 ± 0.21	2.07 ± 0.2	0.002
Ca^1mo^ <2.07	23 (21.9)	14 (60.9)	<0.001
Ca^1mo^ ≥2.07	82 (78.1)	9 (39.1)	
Ca^end^	2.14 ± 0.17	2.00 ± 0.23	0.002
Serum PTH (pmol/L)			
PTH preop	5.04 ± 2.11	6.34 ± 5.7	0.293
PTH^24h^ postop	0.98 ± 1.30	0.98 ± 1.38	0.995
PTH^1mo^ postop	1.45 ± 0.56	1.20 ± 0.67	0.067
PTH^1mo^ <1.2	28 (26.7)	17 (73.9)	<0.001
PTH^1mo^ ≥1.2	77 (73.3)	6 (26.1)	
PTH^end^	2.47 ± 0.99	1.03 ± 0.25	<0.001
Months till recovery	8.23 ± 13.06	–	–
Follow-up (months)	17.00 ± 18.04	28.39 ± 16.98	0.007

Continuous variables given as mean ± standard deviation; preop, preoperative; postop, postoperative; h, hours; mo, month.

**Table 3:** Multivariate analysis for predicting permanent hypoparathyroidism in patients with protracted hypoparathyroidism.

Variable	OR	95% CI	*P*
Carbon nanoparticles	0.280	0.082 - 0.957	0.042
Parathyroid autotransplanted	–	–	–
1 gland	0.320	0.093 - 1.099	0.070
2-3 glands	0.143	0.027 - 0.767	0.023
Calcuim 1^mo^> 2.07 mmol/L	0.217	0.070 - 0.678	0.009
PTH 1^mo^> 1.2 pmol/L	0.106	0.032 - 0.353	<0.001

OR, odds ratio; CI, confidence interval.

### Time of Recovery of Parathyroid Function

**Figure 2:**
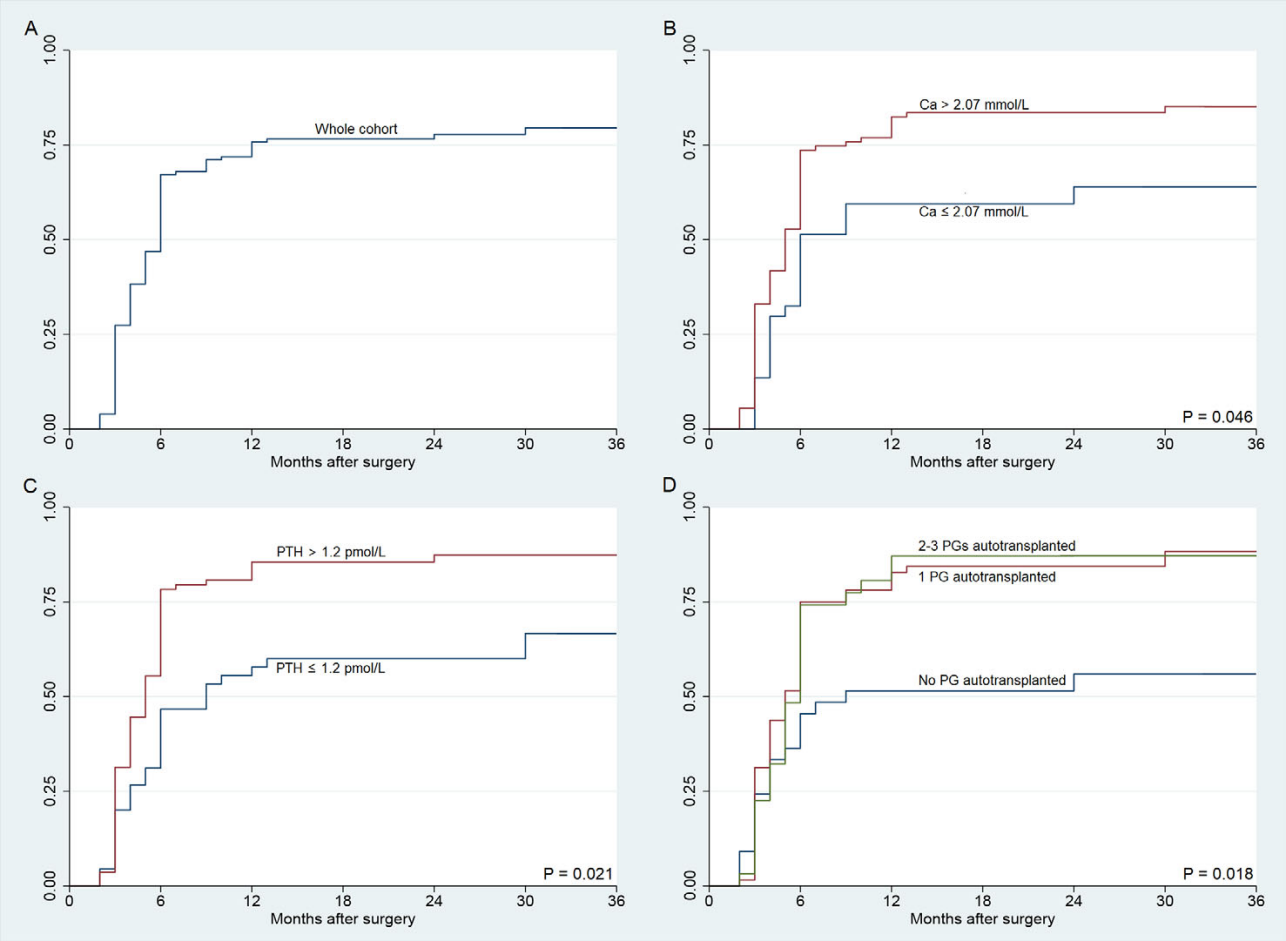
Time to recovery of the parathyroid function in patients with protracted hypoparathyroidism. **(A)** Time to recovery of the parathyroid function for the whole cohort (n = 128). **(B)** Recovery of the parathyroid function according to serum calcium concentration at one month. **(C)** Recovery of the parathyroid function according to parathormone at one month. **(D)** Recovery of the parathyroid function according to parathyroid glands autotransplantation. Horizontal axis uses logarithmic scale (months). *P* values from the Log Rank (Mantel–Cox) test.

No significant clinical variables were associated with recovery of parathyroid function within or after six months (**Tables 4** and **5**). Early recovery of parathyroid function (<6 months) occurs in 81.9% (86/105) patients, but 18.1% (19/105) patients recover after 6 months. The timing of parathyroid function recovery was shown in **Figure 2**. Factors significantly favoring early recovery of parathyroid function were serum calcium levels >2.07 mmol/L (Hazard ratio [HR], 1.628; 95% confidence interval [CI], 1.009 – 2.628; *P* = 0.046) and PTH > 1.2 pmol/L at 1 month (HR, 1.702; 95% CI, 1.083 – 2.628; *P* = 0.021). The number of autotransplanted parathyroid glands was associated with recovery of parathyroid function (HR, 1.399; 95% CI, 1.060 – 1.846; *P* = 0.018).

**Table 4:** Demographic and disease-related variables of patients recovering from protracted hypoparathyroidism within or beyond six months after surgery.

Variables	≤6 months N = 86 (%)	>6 months N = 19 (%)	*P*
Gender			0.542
Male	22 (25.6)	3 (15.8)	
Female	64 (74.4)	16 (84.2)	
Age, years	41.5 ± 12.4	39.6 ± 10.2	0.479
≤55*	75 (87.2)	19 (100)	0.208
>55*	11 (12.8)	0 (0)	
BMI	22.60 ± 3.56	22.87 ± 3.28	0.750
Hypertension**			1.000
No	79 (91.9)	18 (94.7)	
Yes	7 (8.1)	1 (5.3)	
Diabetes			1.000
No	84 (97.7)	18 (94.7)	
Yes	2 (2.3)	1 (5.3)	
Hashimoto’s thyroiditis			0.777
No	66 (76.7)	14 (73.7)	
Yes	20 (23.3)	5 (26.3)	
Hypothyroidism**			1.000
No	84 (97.7)	18 (94.7)	
Yes	2 (2.3)	1 (5.3)	
^131^I ablation			0.461
No	35 (40.7)	6 (31.6)	
Yes	51 (59.3)	13 (68.4)	
T stage			0.446
T1a	22 (25.6)	5 (26.3)	
T1b	24 (27.9)	9 (47.4)	
T2	5 (5.8)	1 (5.3)	
T3a	1 (1.2)	0 (0)	
T3b	22 (25.6)	2 (10.5)	
T4a	11 (12.8)	1 (5.3)	
T4b	1 (1.2)	1 (5.3)	
N stage			0.058
N0	42 (48.8)	6 (31.6)	
N1a	18 (20.9)	9 (47.4)	
N1b	26 (30.2)	4 (21.1)	

Continuous variables given as mean ± standard deviation; *Fisher’s test.; **χ^2^ test with continuity correction.

**Table 5:** Surgical and laboratory variables of patients recovering from protracted hypoparathyroidism within or beyond six months after surgery.

Variables	≤6 months N = 86 (%)	>6 months N = 19 (%)	*P*
Carbon nanoparticles			1.000
No	20 (23.3)	5 (26.3)	
Yes	66 (76.7)	14 (73.7)	
Central neck dissection			0.209
No	16 (18.6)	6 (31.6)	
Yes	70 (81.4)	13 (68.4)	
Central neck dissection (Yes)			0.408
Unilateral	19 (27.1)	5 (38.5)	
Bilateral	51 (72.9)	8 (61.5)	
Lateral neck dissection**			0.371
No	56 (65.1)	15 (78.9)	
Yes	30 (34.9)	4 (21.1)	
No. of autotransplanted parathyroid glands			0.170
0	15 (17.4)	7 (36.8)	
1	48 (55.8)	8 (42.1)	
2-3	23 (26.7)	4 (21.1)	
Serum calcium (Ca, mmol/L)			
Ca preop	2.32 ± 0.09	2.31 ± 0.09	0.565
Ca^24h^ postop	2.03 ± 0.21	1.99 ± 0.14	0.410
Ca^1mo^ postop	2.22 ± 0.22	2.19 ± 0.18	0.580
Ca^1mo^ <2.07**	19 (22.1)	4 (21.1)	1.000
Ca^1mo^ ≥2.07**	67 (77.9)	15 (78.9)	
Ca^end^	2.15 ± 0.15	2.10 ± 0.25	0.393
Serum PTH (pmol/L)			
PTH preop	5.13 ± 2.16	4.63 ± 1.90	0.318
PTH^24h^ postop	0.94 ± 1.35	1.14 ± 1.09	0.479
PTH^1mo^ postop	1.45 ± 0.53	1.47 ± 0.68	0.901
PTH^1mo^ <1.2	21 (24.4)	7 (36.8)	0.268
PTH^1mo^ ≥1.2	65 (75.6)	12 (63.2)	
PTH^end^	2.56 ± 1.00	2.09 ± 0.86	0.049
Months till recovery	4.27 ± 1.37	26.16 ± 23.71	0.001
Follow-up (months)	14.92 ± 15.60	27.29 ± 25.21	0.067

Continuous variables given as mean ± standard deviation; **χ^2^ test with continuity correction; preop, preoperative; postop, postoperative; h, hours; mo, month.

## Discussion

Since the period of parathyroid function recovery and its influencing factors are still unclear, the definition of permanent hypoparathyroidism is still under exploration. More and more evidence has indicated that recovery of parathyroid function is a dynamic process that may occur for a long time after surgery (16, 17). Our study followed patients for an average of 28 months to diagnose permanent hypoparathyroidism and confirmed that prolonged follow-up of patients with protracted hypoparathyroidism is necessary. If 6 months after surgery were used as the cut-off time, approximately 45.2% (19/42) patients would be over diagnosed as permanent hypoparathyroidism.

Some studies have found that parathyroid function can take longer than 6 months or even more than 1 year to recover. Villarroya-Marquina et al. included 142 patients with protracted hypoparathyroidism at 1 month after surgery and found 106 (75%) of them recovered parathyroid function during the follow-up. Of the 106 patients, 73 (69%) patients recovered parathyroid function within 6 months, 21 (19.8%) patients within 1 year, and 12 (11.3%) patients over 1 year (17). Kim et al. studied 1467 patients with total thyroidectomy and found 22 (1.5%) patients were diagnosed with permanent hypoparathyroidism at 6 months after surgery, but of these, 5 (0.3%) patients regained parathyroid function during the follow-up (18). Ritter et al. followed 1054 patients after total thyroidectomy, of whom 189 (17.9%) patients had transient hypoparathyroidism; 132 (69.8%) of the patients recovered within 2 months, 9 (4.7%) recovered between 6 and 12 months and 20 (10.6%) patients showed permanent hypoparathyroidism (6). However, Ritter et al. only followed up to one year and didn’t distinguish protracted and transient hypoparathyroidism.

Factors that positively influenced the recovery of parathyroid function were mostly surgery itself. Use of carbon nanoparticles could prevent parathyroid glands from direct injuries by accurate identification of the parathyroid glands, which was a prerequisite for parathyroid function preservation. A meta-analysis in 2020 demonstrated that administrating carbo nanoparticles was associated with a lower incidence of accidental gland removal (OR, 0.28; 95% CI, 0.21 to 0.37; P<0.01) and lower rates of transient hypoparathyroidism (OR, 0.46; 95% CI, 0.33 to 0.64; P <0.01) (14). In addition, we found the number of autotransplanted parathyroid glands was associated with recovery of parathyroid function. El-Sharaky et al. considered that postoperative parathyroid function of autotransplanted parathyroid pieces would recover gradually from the 2 to 6 weeks, following blood tests and electron microscopy analysis (19). Therefore, autotransplanted parathyroids could help the early recovery of function for patients with protracted hypoparathyroidism, theoretically. However, in our center, the parathyroids were autotransplanted into contralateral sternocleidomastoid muscle, which did not allow a direct check of graft vitality. Cavallaro et al. put forward reimplanting the parathyroids into one side forearm subcutaneous tissue, which could directly check the graft function by comparing serum PTH levels of the two arms (20). Besides, they also found only 88% (22/25) patients recovered parathyroid function in 1 month and 96% (24/25) recovered within 3 months (20). In our study, no parathyroid autotransplantation showed less effect for parathyroid function recovery, and we conjectured it might due to intraoperative mechanical or thermal injury to parathyroids preserved *in situ* which only looked intact but dysfunction for inadequate blood supply or slow fibrosis. It inherently less likely for patients with all functional parathyroid glands *in situ* to develop protracted hypoparathyroidism, therefore, patients with protracted hypoparathyroidism were of higher possibility of injured parathyroid glands, which led to the lower incidence of parathyroid function recovery. However, it was unknown whether some part of the protract hypoparathyroidism was some kind due to parathyroid autotransplantation, and therefore it was not sufficient to determine the role of parathyroid autotransplantation on the overall cohort.

Biochemical variables at 24 hours postoperatively were unable to predict permanent hypoparathyroidism, emphasizing that recovery of parathyroid function is a long and dynamic process (21). Low serum PTH concentrations in the immediate postoperative period are a good marker for the development of postoperative hypocalcemia; however, its ability to predict long-term or permanent hypoparathyroidism is very limited (16, 22). While biochemical variables at where protracted hypoparathyroidism was diagnosed were of some value in our study. Patients with relatively high 1-month serum concentrations of PTH (>1.2 pmol/L) recovered at a higher rate and faster than patients with lower PTH levels. The similar association was seen in patients with higher calcium levels (>2.07 mmol/L), which positively influenced recovery of parathyroid function. One theory holds that serum calcium concentrations in the upper normal range put at rest the injured or ischemic parathyroid glands and allow for a better recovery of the remaining viable parathyroid parenchyma from intraoperative injury (23). We assumed the serum calcium concentrations at 1 month were mostly from the calcium and calcitriol supplementation, and this could validate the above theory. However, Villarroya-Marquina et al. found no correlation between serum calcium and PTH and patients with more elevated serum calcium concentrations recovered early than those with mild hypercalcemia (17). Further studies are needed to clarify this multifaceted physiological riddle.

A limitation of the study is that our data focused on papillary thyroid carcinoma and cannot expand to other thyroid diseases, because the proportion of other thyroid diseases is too small in our center. That a few patients recovered parathyroid function during the long term (over 6 months or even 1 year) made it difficult to further identify predictors between permanent hypoparathyroidism and these patients, and this is the other limitation.

In conclusion, recovery of parathyroid function will mostly depend on the serum calcium and PTH concentrations achieved and it is suggested that the average patient with hypocalcemia after total thyroidectomy should be discharged on calcium and calcitriol to maintain serum calcium concentrations of at least 2.07 mmol/L during the first month. Permanent hypoparathyroidism should not be diagnosed easily by time after surgery, since up to one-fifth of the patients will recover beyond 6 months period and a few even after one year.

## Data Availability Statement

The raw data supporting the conclusions of this article will be made available by the authors, without undue reservation.

## Ethics Statement

Written informed consent was obtained from the individual(s) for the publication of any potentially identifiable images or data included in this article.

## Author Contributions

YQ and AS conceived and designed the study. YQ, ZX and QX executed the study. YQ, QY and YL analyzed and involved in interpretation of data. YQ, ZX and AS drafted the article. YQ, YL and AS made final approval of the version to be published. All authors contributed to the article and approved the submitted version.

## Ethics Approval and Consent to Participate

The study was approved by the ethic committee of West China Hospital, Sichuan University. Informed consent was obtained.

## Funding

This work was supported by the 1.3.5 project for disciplines of excellence, West China Hospital, Sichuan University (ZY2017309).

## Conflict of Interest

The authors declare that the research was conducted in the absence of any commercial or financial relationships that could be construed as a potential conflict of interest.
